# Strategies for Treating Latent Multiple-Drug Resistant Tuberculosis: A Decision Analysis

**DOI:** 10.1371/journal.pone.0030194

**Published:** 2012-01-17

**Authors:** David P. Holland, Gillian D. Sanders, Carol D. Hamilton, Jason E. Stout

**Affiliations:** 1 Department of Medicine, Duke University Medical Center, Durham, North Carolina, United States of America; 2 Division of Infectious Disease, Duke University Medical Center, Durham, North Carolina, United States of America; 3 FHI 360, Durham, North Carolina, United States of America; Fundació Institut d'Investigació en Ciències de la Salut Germans Trias i Pujol. Universitat Autònoma de Barcelona. CIBERES, Spain

## Abstract

**Background:**

The optimal treatment for latent multiple-drug resistant tuberculosis infection remains unclear. In anticipation of future clinical trials, we modeled the expected performance of six potential regimens for treatment of latent multiple-drug resistant tuberculosis.

**Methods:**

A computerized Markov model to analyze the total cost of treatment for six different regimens: Pyrazinamide/ethambutol, moxifloxacin monotherapy, moxifloxacin/pyrazinamide, moxifloxacin/ethambutol, moxifloxacin/ethionamide, and moxifloxacin/PA-824. Efficacy estimates were extrapolated from mouse models and examined over a wide range of assumptions.

**Results:**

In the base-case, moxifloxacin monotherapy was the lowest cost strategy, but moxifloxacin/ethambutol was cost-effective at an incremental cost-effectiveness ratio of $21,252 per quality-adjusted life-year. Both pyrazinamide-containing regimens were dominated due to their toxicity. A hypothetical regimen of low toxicity and even modest efficacy was cost-effective compared to “no treatment.”

**Conclusion:**

In our model, moxifloxacin/ethambutol was the preferred treatment strategy under a wide range of assumptions; pyrazinamide-containing regimens fared poorly because of high rates of toxicity. Although more data are needed on efficacy of treatments for latent MDR-TB infection, data on toxicity and treatment discontinuation, which are easier to obtain, could have a substantial impact on public health practice.

## Introduction

Although the incidence of multiple-drug resistant tuberculosis (MDR-TB, defined as resistance to isoniazid plus rifampin) in countries with developed market economies is low, MDR-TB incidence is high in many parts of the world [1]. The relative ease of travel between these countries and countries with low TB incidence facilitates the spread of transmissible diseases such as MDR-TB. Therefore, the development of strategies to contain the spread of MDR-TB is important not only for the developing world but for countries with low MDR-TB incidence as well.

One such strategy is the prevention of active MDR-TB through effective treatment during the latent phase of TB infection (LTBI). Unfortunately, no treatment regimens for latent MDR-TB infection have been tested in a randomized, controlled human trial [2]. Based on animal models, case series, and expert opinion, two preventive regimens are currently recommended by the U.S. Centers for Disease Control and Prevention (CDC): Pyrazinamide plus ethambutol or pyrazinamide plus a fluoroquinolone (such as moxifloxacin), each for 6–12 months [3]. However, both regimens have high rates of toxicity [4]–[7]. For immunocompetent individuals, careful follow-up without treatment is also considered a reasonable option. Therefore, the question of how to treat latent MDR-TB infection (MDR-LTBI) – or even whether to treat it – remains controversial.

Part of the solution may be found in new drugs currently in development for the treatment of active TB. One of these drugs, PA-824, has excellent sterilizing activity [8] and would therefore be expected to have utility in the treatment of LTBI. Furthermore, because of its novel mechanisms of action [9], it has activity against MDR-TB [10]. The role of this and other new drugs in treating latent MDR-TB infection is an area of much interest.

To help answer these questions about current and future treatment of latent MDR-TB infection, Nuermberger and colleagues employed a murine model of arrested TB to compare several different regimens for the treatment of MDR-LTB [11]. Although the mouse model is an imperfect representation of human LTBI, the model has successfully identified two regimens for treating drug-susceptible LTBI, rifampin plus pyrazinamide and isoniazid plus rifapentine, which have proven efficacious in clinical trials [12]–[14].

Anticipating clinical trials based on similar murine models, we sought to define the key parameters that would make a future regimen potentially an effective and cost-effective strategy. Such parameters and the impact of the uncertainty underlying their estimates will be important for designing clinical trials to test potential regimens. Therefore, we designed a mathematical model to compare the expected performance of six potential regimens over a range of clinically plausible estimates to provide useful information to public health practitioners in the treatment of latent MDR-TB infection.

## Materials and Methods

**Figure 1:**
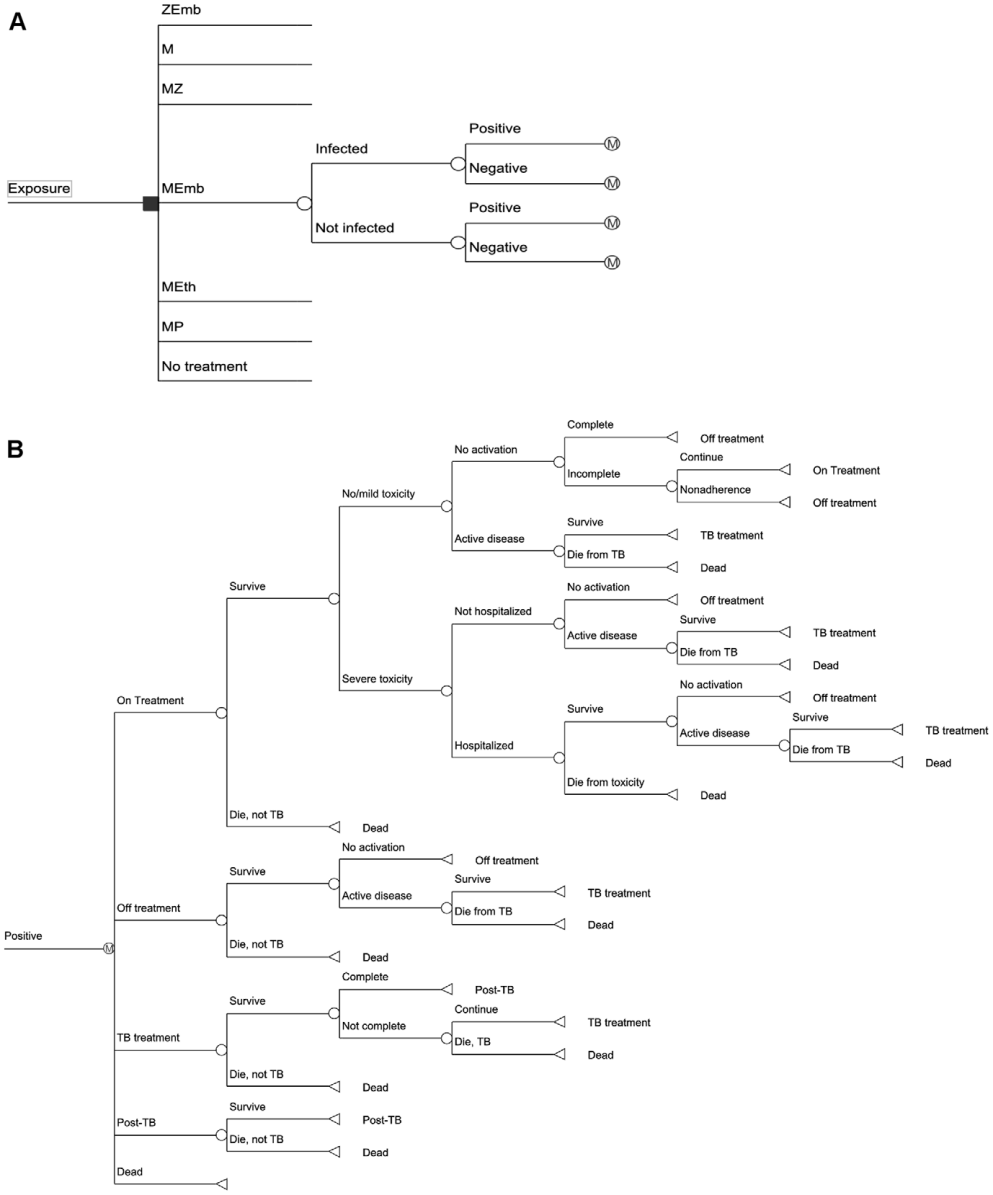
Schematic of decision tree. A. Primary tree showing decision point between regimens and probability of a positive or negative test given presence or absence of infection. B. Positive test subtree. Individuals begin “on treatment” then move to “off treatment” due to toxicity, non-adherence, or treatment completion. Patients in “off treatment” can develop active disease and move to “TB treatment;” after treatment for active disease, they move to “prior TB.” Age-related mortality and death from TB or toxicity is also included. The negative test subtree is similar to the positive test subtree without the “on treatment” branch. Abbreviations: MZ = moxifloxacin+pyrazinamide, ZEmb = pyrazinamide+ethambutol, MEth = Moxifloxacin+ethionamide, MP = moxifloxacin+PA-824, M = moxifloxacin monotherapy.

Using TreeAge Pro (release 1.0.2, 2009, TreeAge Software, Inc., Williamstown, MA), we created a Markov model [15] (**Figure 1**) of a hypothetical exposure of MDR-TB in a cohort of individuals otherwise at low risk for TB. We compared six different treatment regimens (plus a strategy of “no treatment”):

1. Pyrazinamide 1,500 mg plus ethambutol 1,600 mg (ZEmb)
2. Moxifloxacin 400 mg monotherapy (M)
3. Moxifloxacin 400 mg plus pyrazinamide 1,500 mg (MZ)
4. Moxifloxacin 400 mg plus ethambutol 1,600 mg (MEmb)
5. Moxifloxacin 400 mg plus ethionamide 1,000 mg (MEth)
6. Moxifloxacin 400 mg plus PA-824 400 mg (MP)
7. No treatment

Infecting MDR organisms were assumed to be susceptible to all drugs in the model.

To account for excess cost associated with treating individuals testing falsely positive on available diagnostic tests, we assumed that only a portion of the cohort became infected after exposure (10% in the base-case, see below). A test for LTBI was then applied, the sensitivity and specificity of which was varied to simulate either tuberculin skin testing (TST) or interferon gamma release assay testing. Individuals were either treated or not treated based on the results of this test. Because individuals in the cohort were assumed to be at low risk for prior TB infection, all active cases were assumed to be multi-drug resistant.

The model was analyzed from the societal perspective using cycles of one month duration. Payoffs were calculated for costs and quality-adjusted life-years (QALY) based on U.S. data. Costs and life-years were discounted at 3%. Incremental cost-effectiveness ratios (ICER) were calculated for each regimen, with a willingness-to-pay threshold of $50,000 per QALY chosen to determine cost-effectiveness. All costs were based on U.S. public health pricing and were converted to 2009 U.S. dollars using the Gross Domestic Product deflator [16].

### Transition probabilities

The initial probabilities and utility adjustments used in the model are shown in **Tables 1** and **2**.

**Table 1:** Model parameters and utility adjustments, point estimates and ranges for sensitivity analyses.

Variable	Estimate	Range	Source
Lifetime risk of TB	0.04	0.04–0.06	[3], [17]
Proportion of patients hospitalized for toxicity	0.1	0.05–0.2	[18]
Proportion of hospitalized patients dying from drug toxicity	0.01	0–0.03	[18]
Probability of death from TB	0.12	0–0.12	[19]
Number of secondary cases per active case	0.4	0–1.2	[20], [21]
Initial probability of infection	0.1	0–1	(assumed)
Test characteristics for detection of latent TB			
Sensitivity	0.78	0.63–0.82	[22]
Specificity	0.95	0.59–0.99	[22]
Quality of life adjustments (life-years)			
LTBI treatment	0.97	0.85–0.97	[23]
Treatment-limiting toxicity	0.75	0.65–085	(assumed)
Hospitalization	0.5	0.40–0.60	(assumed)
Treatment of active TB	0.90	0.64–0.93	[23]
Prior TB	0.95	0.85–1	(assumed)

**Table 2:** Base-case estimates for protection, adherence, and toxicity for the six regimens and ranges for sensitivity analyses.

Variable	Estimate	Range	Source
Risk reduction from treatment			
Pyrazinamide+ethambutol (ZEmb)	0.62	0–1	[11]
Moxifloxacin+pyrazinamide (MZ)	0.90	0–1	[11]
Moxifloxacin monotherapy (M)	0.62	0–1	[11]
Moxifloxacin+ethambutol (MEmb)	0.76	0–1	[11]
Moxifloxacin+ethionamide (MEth)	0.69	0–1	[11]
Moxifloxacin+PA-824 (MP)	0.98	0–1	[11]
Non-adherence per month (no toxicity)			
0–4 months	0.05	0–1	[4], [5], [24]–[26]
5–6 months	0.08	0–1	
Probability of stopping from toxicity			
Pyrazinamide+ethambutol (ZEmb)	0.58	0–1	[7]
Moxifloxacin+pyrazinamide (MZ)	0.67	0–1	[4]–[6], [27]
Moxifloxacin monotherapy (M)	0.014	0–1	(assumed)
Moxifloxacin+ethambutol (MEmb)	0.04	0–1	(assumed)
Moxifloxacin+ethionamide (MEth)	0.07	0–1	[19]
Moxifloxacin+PA-824 (MP)	0.04	0–1	(assumed)

To determine the probability of infection after exposure, we started with North Carolina TB surveillance data (Aggregate Reports for Tuberculosis Program Evaluation, unpublished). For 2009, 16% of close contacts to active cases had a positive TST. However, because over 4% of individuals in the U.S. have a positive TST at baseline [28], we assumed a probability of new infection of 10%.

For recently-infected contacts to an active case of drug-susceptible TB, the lifetime risk of progressing from LTBI to active TB is estimated at 7%, with approximately half of the disease occurring in the first two years [3], [17]. However, compared with fully-susceptible strains, drug-resistant strains appear to be somewhat less virulent [29]–[32], generating about half the number of secondary cases [20]. Because contacts to MDR-TB appear to have equal rates of TST positivity [33], the evidence suggests that MDR strains are just as infectious as drug susceptible strains, but patients infected with MDR strains are less likely to activate. Therefore, we adjusted our base-case estimate of lifetime risk down to 4%, while exploring higher rates of activation in the sensitivity analysis.

Members of the MDR-TB-exposed cohort who developed active TB were assumed to infect other individuals, some of whom would eventually develop active TB. A previously-described model of drug-susceptible TB suggests that each case of active TB in the U.S. will result in approximately 1.2 additional future cases through successive transmission distributed over 44 years [21], but due to the lower number of secondary cases generated by MDR strains (as mentioned above), we adjusted the base-case estimate to 0.6. These assumptions were explored in sensitivity analyses.

### Assumptions

We extrapolated initial efficacy estimates from Nuermberger and colleagues' data on murine splenic culture sterilization. In their experiments, the six drug regimens and a control regimen of isoniazid were each given to experimentally-infected mice for six months, after which splenic cultures were obtained. To obtain our estimates, we ranked the regimens in order of their efficacy in the mice, and then divided the regimens into quintiles. Using isoniazid as a reference, we extrapolated the efficacy of each quintile, assuming that all regimens had at least partial efficacy. The estimates used in the model are listed in **Table 1** and explored in sensitivity analyses.

We assumed that the risk of treatment-limiting adverse events for moxifloxacin was the same as for other quinolones. Under this assumption, data on the tolerability of pyrazinamide/ethambutol and moxifloxacin/pyrazinamide was taken directly from available literature [4]–[7], [27]. Data suggest that there is little additional toxicity when fluoroquinolones are given as part of a larger TB treatment regimen [19], [34] and fluoroquinolone monotherapy appears to be relatively well-tolerated [35], so we assumed that moxifloxacin monotherapy has a toxicity profile similar to isoniazid. No data for tolerability of moxifloxacin/ethambutol or moxifloxacin/ethionamide exist, so we used toxicity rates published for ethambutol and ethionamide in active MDR-TB to estimate their additional toxicity [19]. Likewise, no data on the administration of PA-824 beyond seven weeks has been published [36], but we assumed in our base-case analysis that it had a similar tolerability profile to moxifloxacin monotherapy.

Although no cases series for treatment of MDR-LTBI have reported toxicity resulting in death, numbers are quite low. Based on data from isoniazid monotherapy, we assumed that 1% patients who experience treatment-limited toxicity would require hospitalization and that of those patients, 1% would die [18]. These toxicity estimates are shown in **Table 2**.

Case series suggest that the mortality of active MDR-TB is approximately 12% [19]. We assumed that half of these patients would die within the first month of treatment of active disease [37], with the balance distributed throughout the remainder of treatment. The risk of non-TB death was taken from age-specific mortality tables [38].

In case series of pyrazinamide-containing regimens, some individuals discontinued medications prematurely but did not experience serious side effects. Although low numbers preclude an accurate estimate, the proportion of patients in these series who stopped due to non-adherence appears to be similar to non-completion rates reported in studies of isoniazid. Therefore, we assumed that 80% of individuals not experiencing toxicity would complete four months of each regimen and 64% would complete six months [24], [25]. Therefore, 5% of individuals would discontinue therapy each month for the first four months and 8% would discontinue therapy each month thereafter (**Table 2**). We assumed that these individuals were lost to follow up and did not restart treatment [25].

Because some health states in our model are associated with lower quality of life, we included utility adjustments to life-years for patient transitioning through these health states (shown in **Table 1**). Data for these adjustments are limited [23], so we tested a wide range of assumptions in sensitivity analyses.

### Cost estimates

Costs for treatment of latent infection are shown in **Table 3**; because we assumed that all patients within each group were tested, costs of testing were not included. Drug prices were taken from current North Carolina public health pharmacy costs; PA-824 was assumed to cost the same as moxifloxacin. It was assumed that all patients on a pyrazinamide-containing regimen and 40% of patients on other regimens would receive monthly laboratory monitoring. Costs associated with severe toxicity and hospitalization were incurred only for patients experiencing those events.

**Table 3:** Costs for treating latent MDR-TB infection, point estimates and ranges for sensitivity analyses.

	Estimate	Range	Reference
Monthly visit costs			
Nursing visit ($31.97/hr)	$17.41		[39]
Labs*	$7.70		[39]
Physician visit**	$11.90		[39]
Total visit cost	$37.01	$27.76–46.26	
Drug costs			
Moxifloxacin 400 mg	$3.42		***
Pyrazinamide 500 mg	$0.43		***
Ethambutol 400 mg	$0.46		***
Ethionamide 250 mg	$2.10		***
PA-824	$3.42		(assumed)
Total monthly costs			
Pyrazinamide+ethambutol	$129.41		
Moxifloxacin+pyrazinamide	$190.61		
Moxifloxacin monotherapy	$140.12		
Moxifloxacin+Ethambutol	$181.52		
Moxifloxacin+Ethionamide	$403.91		
Moxifloxacin+PA-824	$345.32		
Severe toxicity costs			
Lab monitoring	$161.60		[39]
Hospitalization (7 days)	$5428.78	$4,071–$6,786	[40]

*Average cost of routine monitoring and evaluation for mild toxicity assuming 40% of patients will require monthly monitoring of hepatic enzymes (100% for patients on pyrazinamide) and 1.4% will have toxicity severe enough to require a physician evaluation but that does not limit treatment.

**Medicare cost assumed for 15 minutes of physician time for 40% of patients.

***North Carolina Public Health Pharmacy Data.

Costs for treatment of active disease are shown in **Table 4**. We assumed that 75% of patients with active disease would be hospitalized, following which patients would receive directly-observed therapy (standard of care for patients with MDR-TB) every day for a total of 18 months. We assumed patients would come to the clinic for intramuscular injections during the first four months of therapy but would receive directly-observed therapy (DOT) in the field thereafter.

**Table 4:** Costs for treating active MDR-TB, point estimates and ranges for sensitivity analyses.

	Units	Cost/unit	Total cost	Range	Reference
Diagnosis			$456.84	$342–571	[41]
Inpatient treatment			$24,853	$5,278–73,572	[42]
DOT costs					[41], [43]
Outreach worker (per hour)	1	$17.10	$17.10		
Patient time (per hour)	0.1	$15.16	$1.52		
Driving (miles)	10	$0.55	$5.50		
Total DOT cost			$24.12	$18–30	
Outpatient (per month, months 1–4)					
Drug	20	$30.55	$611.00		Table 2
DOT	0	$22.32	0		
Nursing (30 minutes)	10	$32.62	$326.20		[41]
Pt. time per monthly visit	1.5	$15.16	$454.80		[41]
Pt. travel (10 miles @ $0.55/mile)	20	$110.00	$2,200.00		[41]
Monitoring^*^	4	$90.32	$361.28		[41]
Total outpatient (months 1–4)			$3,953.28	$2,964–4,941	
Outpatient (per month, months 5–18)					
Drug	20	$25.04	$500.80		Table 2
DOT	20	$24.12	$482.32		
Nursing (30 minutes)	0.5	$32.62	$16.31		[41]
Pt. time per monthly visit	1.5	$15.16	$22.74		[41]
Monitoring*	1	$90.32	$36.13		[41]
Total outpatient treatment (mos. 5–18)			$1,112.49	$834–1,390	
Contact tracing/testing (90% of contacts screened)	5.2	$135.71	$635.14	$478–794	[43], [44]
Total per case – 18 months therapy			$51,220.22		

### Sensitivity analyses

To account for parameter uncertainty, we conducted multiple sensitivity analyses by varying the parameter estimates over the specified ranges. Where available, we used ranges for inputs based on published literature. For costs, we determined the range by adding or subtracting 25% from the base case estimate. One-, two-, and three-way sensitivity analyses were performed as appropriate. Our primary analysis focused on the search for thresholds that could define clinical trial parameters, primarily efficacy and toxicity for each regimen, so estimates for these inputs were tested over a wide range.

## Results

**Figure 2:**
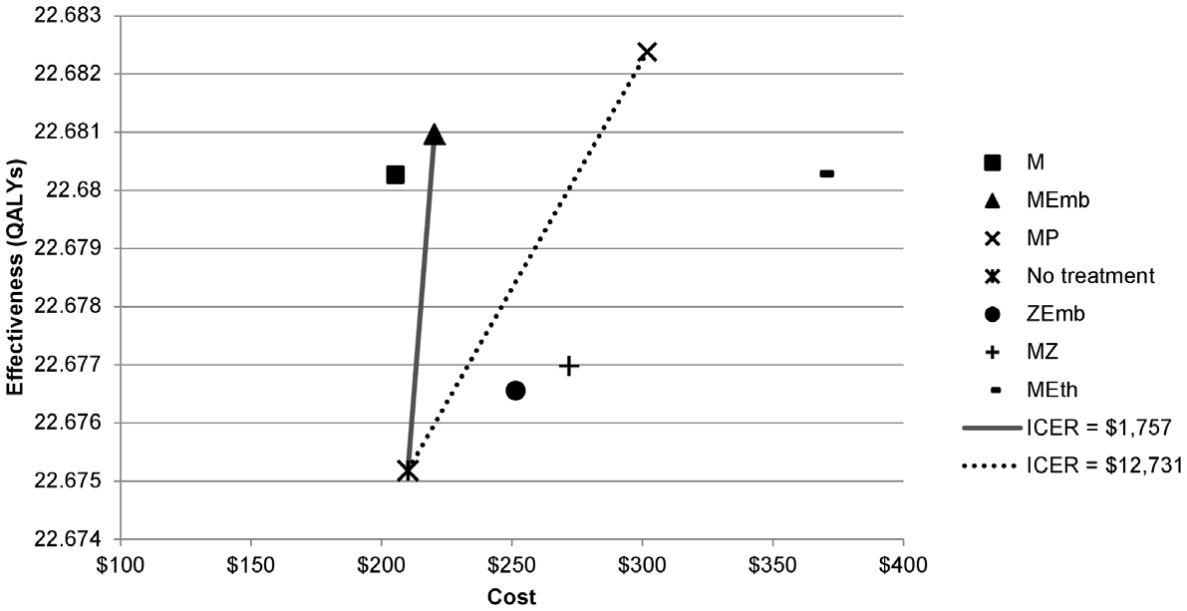
Cost-effectiveness plot for six regimens plus the “no treatment” strategy. Superior regimens are lower in cost (toward the left) and greater in efficacy (toward the top). Incremental cost-effectiveness ratios (ICERs) are shown for the two most effective regimens referenced to the strategy of “no treatment.” *Abbreviations: MZ = moxifloxacin/pyrazinamide, ZEmb = pyrazinamide/ethambutol, MEth = moxifloxacin/ethionamide, MP = moxifloxacin/PA-824, M = moxifloxacin monotherapy. ICER = incremental cost-effectiveness ratio in dollars per quality-adjusted life-year*.

The point estimates for costs, QALY's, and ICER's for each regimen are shown in **Tables 5** and **6**, and **Figure 2**. In the base case, all regimens were associated with greater quality-adjusted life expectancy than the “no treatment” strategy. Furthermore, the “no treatment” strategy was dominated by (more expensive and less effective than) moxifloxacin monotherapy.

**Table 5:** Lifetime costs and quality-adjusted life-years (QALY) for all regimens, efficacy predicted by murine model: MP>MZ>>MEmb>MEth = Isoniazid>M = ZEmb.

Strategy	Cost	Incremental cost	Effectiveness (QALY)	Incremental effectiveness	ICER ($/QALY)*
M	$205.32	(ref)	22.68017	(ref)	
No treatment	$210.08	$4.75	22.67518	(0.00509)	(Dominated)
MEmb	$220.25	$14.93	22.68097	0.000702	$21,253
ZEmb	$251.36	$31.10	22.67656	(0.00441)	(Dominated)
MZ	$271.85	$51.60	22.678698	(0.00399)	(Dominated)
MP	$301.74	$81.49	22.68238	0.00141	$57,771
MEth	$368.60	$66.86	22.68029	(0.00209)	(Dominated)

Regimens referenced to the lowest-cost strategy.

*Incremental cost-effectiveness ratios (ICERs) are calculated relative to next-lowest-effectiveness option.

*Abbreviations: MZ = moxifloxacin+pyrazinamide, ZEmb = pyrazinamide+ethambutol, MEth = Moxifloxacin+ethionamide, MP = moxifloxacin+PA-824, M = moxifloxacin monotherapy*.

**Table 6:** Lifetime costs and quality-adjusted life-years (QALY) for all regimens, efficacy predicted by murine model: MP>MZ>>MEmb>MEth = Isoniazid>M = ZEmb.

Strategy	Cost	Incremental Cost^1^	Effectiveness (QALY)	Incremental effectiveness^1^	ICER ($/QALY)^1^
No treatment	$210.08	(ref)	22.67518	(ref)	
ZEmb	$251.36	$41.28	22.67656	0.001381	$29,891.72
MZ	$271.85	$61.77	22.67698	0.001802	$34,280.37
M	$205.33	($4.75)	22.68026	0.005087	(Dominant)
MEth	$368.60	$158.52	22.68029	0.005113	$31,003.29
MEmb	$220.25	$10.18	22.68097	0.00579	$1,757.49
MP	$301.74	$91.66	22.68238	0.0072	$12,730.82

Regimens referenced to a strategy of “no treatment” and ordered by increasing effectiveness.

1 Incremental costs, effectiveness, and cost-effectiveness ratios are referenced to the strategy of “no treatment.”

*Abbreviations: MZ = moxifloxacin+pyrazinamide, ZEmb = pyrazinamide+ethambutol, MEth = Moxifloxacin+ethionamide, MP = moxifloxacin+PA-824, M = moxifloxacin monotherapy*.

Moxifloxacin monotherapy was the lowest cost regimen (due to relatively low treatment costs, efficacy, and tolerability), but moxifloxacin/ethambutol was more effective than this regimen at a cost of $21,252 per QALY. Moxifloxacin/PA-824 was the most effective regimen, though at an ICER of $57,771 per QALY (compared with moxifloxacin/ethambutol), given our cost-effectiveness threshold of $50,000 per QALY it would not be considered a good use of resources. All other regimens were dominated by moxifloxacin/ethambutol.

### Sensitivity Analysis

Both pyrazinamide-containing regimens (moxifloxacin/pyrazinamide and pyrazinamide/plus ethambutol) were substantially less effective than the other drug regimens modeled. Because of the extremely high rates of toxicity for these two regimens, there was no threshold for efficacy of either regimen above which either one became a cost-effective strategy compared with available alternatives. Additionally, they would only become cost-effective compared to moxifloxacin/ethambutol at extremely low estimates for the protection afforded by moxifloxacin/ethambutol combination (<10%).

**Figure 3:**
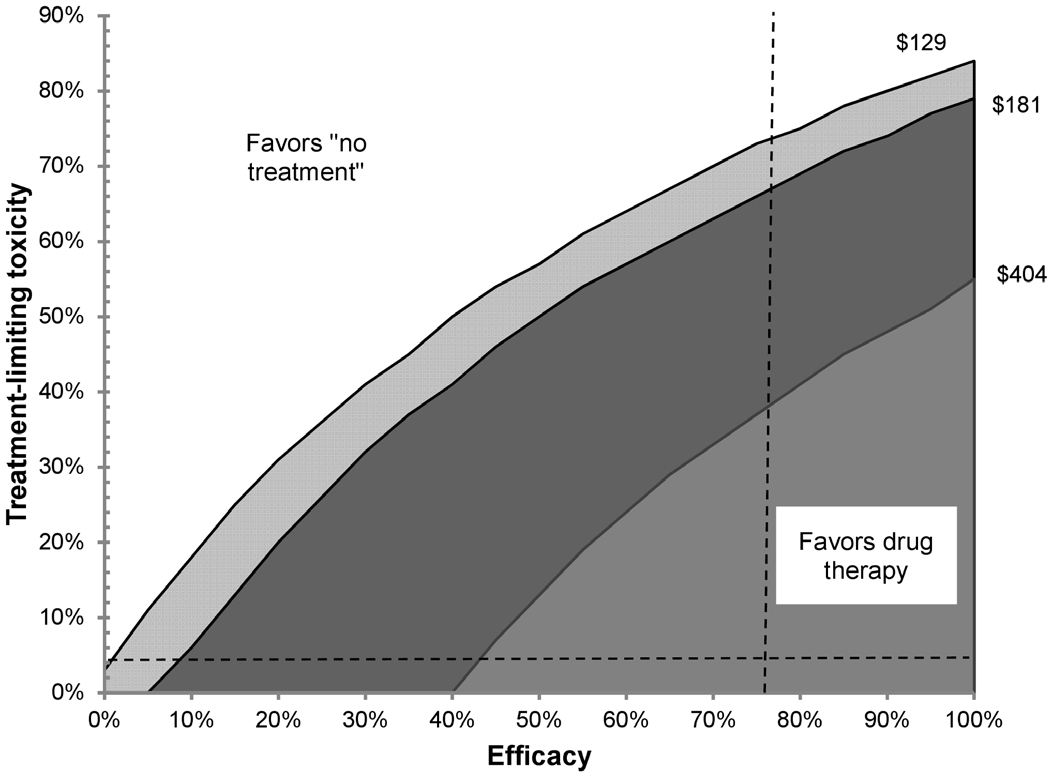
Strategy graph of efficacy vs. toxicity. Solid lines indicate thresholds (“isoclines”) for indifference given monthly drug costs of $129 (pyrazinamide/ethambutol), $181 (moxifloxacin/ethambutol), and $404 (moxifloxacin/ethionamide) per month. Shaded area indicates combinations of toxicity and efficacy for which drug treatment is cost-effective compared to the “no treatment” strategy beneath each of these isoclines. Dotted lines indicate the base-case estimates for efficacy and toxicity of moxifloxacin/ethambutol.

The overall effectiveness of a regimen was determined primarily by its toxicity/tolerability and its efficacy. **Figure 3** shows a strategy graph comparing the moxifloxacin plus ethambutol strategy with the “no treatment” strategies at various drug costs. At base-case estimates for drug costs ($182 per month, equivalent to the cost of moxifloxacin plus ethambutol), the drug regimen could have a relatively low efficacy and still be cost-effective when compared to “no treatment” as long as treatment-limiting toxicity is also low. **Figure 3** also shows thresholds for cost-effectiveness for the least expensive and most expensive drug regimens ($129 and $404 per month, respectively). As an example, the dotted lines on Figure 3 show the point estimates for toxicity and efficacy for moxifloxacin/ethambutol, which would be cost-effective even if the regimen costs were over $404 per month. However, a hypothetical regimen with 60% efficacy and a severe toxicity rate of 50% would only be cost-effective if the cost of treatment were $129 per month (and not at $404 per month).

The cost for PA-824, which is still in development, is unknown, but under base-case estimates for efficacy and tolerability, moxifloxacin/PA-824 would be cost-effective compared to moxifloxacin/ethambutol at a cost per pill for PA-824 of $3.32 or less (compared to the base-case $3.42). Results were not sensitive to variations in the costs of toxicity or TB treatment over the specified ranges.

Results were not sensitive to changes in overall adherence if the adherence rates of all regimens (excluding discontinuation related to toxicity) are similar. Pyrazinamide-containing regimens were not cost-effective even if their adherence rates (not related to toxicity) were 100%.

To account for the variable accuracy of different LTBI tests in different populations, we performed sensitivity analysis on the test characteristics. At a test specificity below 74 (base-case estimate = 95%), none of the drug treatment regimens were cost-effective compared to the “no treatment” strategy. For test specificity between 74–86%, moxifloxacin monotherapy is the preferred strategy. No thresholds for sensitivity were found.

If the prevalence of infection is below 2%, the “no treatment” strategy is preferred over all treatment options. For prevalence between 2–4%, the preferred strategy is moxifloxacin monotherapy.

If the lifetime risk of activation is increased to 6% (baseline estimate = 4%), both moxifloxacin/ethambutol and moxifloxacin dominate the “no treatment” strategy. Furthermore, moxifloxacin/PA-824 becomes cost-effective compared to moxifloxacin/ethambutol at a cost of $33,350 per QALY. Increasing the number of secondary cases of active TB to 1.2 per active case in the cohort (base case estimate = 0.6) also resulted in both moxifloxacin/ethambutol and moxifloxacin dominating the “no treatment” strategy.

The utility of prior TB (base-case estimate 0.95) had one threshold at 0.91 below which moxifloxacin/PA-824 became the preferred strategy. There were no other thresholds for utility adjustment over the specified ranges.

## Discussion

In our analysis, efficacy, toxicity, and cost were the key parameters determining the overall cost effectiveness of the different regimens. Specifically, the tradeoff between efficacy and toxicity was a primary parameter, in that regimens with relatively low efficacy could be cost-effective compared to “no treatment” if they are well-tolerated.

In our base-case analyses, moxifloxacin monotherapy was the lowest cost option, though moxifloxacin/ethambutol was a cost-effective alternative. These strategies maintained superior cost-effectiveness over a wide range of reasonable estimates for adherence and toxicity. The standard regimens recommended by the CDC, pyrazinamide/ethambutol and pyrazinamide/moxifloxacin, while cost-effective compared to the “no treatment” strategy, were substantially less effective than other regimens.

Our model is subject to several limitations. First, many of the parameters in our study are currently unsupported by human clinical trials. However, it was not our attempt to calculate the precise cost-effectiveness for any of the regimens, but rather to determine their *relative* cost-effectiveness over a range of parameter estimates and to determine which uncertainties within the parameter estimates had the greatest impact on our findings and therefore should be prioritized in future research studies.

Next, our model assumes that the MDR-TB isolates are susceptible to most of the second-line antituberculous agents, particularly moxifloxacin. Fluoroquinolone-resistant strains are relatively rare among MDR-TB isolates in the U.S. [45] but are increasingly common worldwide [46], [47]. Although these strains limit the usefulness of our model's most effective regimens outside of the U.S., the results should still be applicable for U.S. TB control programs or other areas where fluoroquinolone resistance is low.

In addition, our analysis was restricted to a population of low-risk individuals. In high-risk individuals, such as those with human immunodeficiency virus infection, even a slight improvement in a regimen's overall effectiveness will prevent a large number of additional cases, magnifying both the health and economic benefit from treatment. In some cases, this additional cost savings may be enough to compensate for a more expensive regimen [48].

Lastly, the results of our model are only applicable to settings with the overall burden of TB is low, such as in most developed economies. We implicitly assumed that no one became re-infected after treatment for LTBI, so any setting where the probability of re-infection is substantial would make all treatments appear less effective [49].

In our analysis, incremental effectiveness was small. There are two primary reasons for this finding. First, our hypothetical cohort was limited to otherwise healthy individuals in a developed economy (e.g., the United States), where mortality from TB is very low, even for MDR-TB. More importantly, however, we modeled exposure and testing in such a way that the majority of individuals in the cohort were not infected and were therefore did not benefit from any treatment. Elimination of this portion of the model increases the incremental effectiveness of the regimens, but also increases costs.

The cost of treating MDR-TB was estimated from local practices and drug costs. Rajbhandary et. al. calculated much higher total costs for treating active MDR-TB (an average of $117,440 in 2011 U.S. dollars per case) [42]. However, much of this higher cost is due to assumptions of greater loss of productivity (including that due to death) than in our model. Furthermore, their study did not describe drug resistance patterns for the patients on which the cost data were based. In an attempt to be conservative, we assumed a “best-case” scenario with limited drug resistance and no requirement for surgery. If these higher estimates were used, all drug regimens would appear much more cost-effective in relation to the “no treatment” strategy.

Of note, analysis of the sensitivity and specificity of our TB testing strategy suggests that the “no treatment” strategy would be preferred in situations where the test specificity is low. In BCG-immunized individuals, the specificity of the tuberculin skin test (approximately 59% [22]) is below our study's threshold for specificity. Therefore, if testing is to be a prelude to treatment, in BCG-vaccinated populations exposed to MDR-TB, a test with higher expected specificity, such as an interferon gamma release assay, would be preferred.

The results of our study are consistent with a prior decision analysis by Stevens and colleagues, who compared a fluoroquinolone plus ethambutol against a strategy of “no treatment” in HIV-uninfected healthcare workers [26] and found that this drug combination was slightly superior to no therapy. However, they did not consider other alternatives, nor did they consider the costs associated with treating TB or secondary cases. Because these costs are an important factor in determining the economic viability of a regimen, their consideration greatly assists public health officials in choosing among available options.

Although data for our assumptions regarding PA-824 are limited, our model suggests it (or other new drugs) is worthy of further study. Assuming a low level of toxicity, it would be expected to be superior to either of the standard pyrazinamide-containing regimens and would be cost-effective compared to the other regimens if its drug costs are lower than estimated.

Clearly, more data are needed to determine the best treatment for latent MDR-TB infection, but even in the absence of such data, our study suggests two important conclusions. First, our analysis shows that regimens need not be highly efficacious to be clinically useful so long as they are well-tolerated. Therefore, in the absence of new efficacy data, a relatively inexpensive study focusing on toxicity and tolerability of either the moxifloxacin/ethambutol or moxifloxacin/PA-824 combinations (or both) would provide important information regarding the utility of these regimens. Second, because toxicity and tolerability were such important determinants of the overall efficacy of the regimens in our model (and are of particular concern for preventive therapy in general), both pyrazinamide-containing regimens performed poorly. Therefore, we believe that the benefit of pyrazinamide-containing regimens for the treatment of latent MDR-TB infection be reconsidered in favor of regimens with better expected tolerability, moxifloxacin monotherapy or moxifloxacin plus ethambutol.
